# On the Use of the Epiglottis

**Published:** 1814-06

**Authors:** 


*For the Medical and Physical Journal.*

### *On the Use of the Epiglottis, by* Mr. Want.

IT may be useful to remark, in reference to the interesting paper of Mr. Magendie on this subject, inserted in the Number for April last, that the opinions contained in it are not new, or peculiar to that gentleman. In one of the early volumes of the *Recueil Periodique*, by Vandermonde, we see the following observations by Tozzetti. "Physiologists have considered the epiglottis to be an organ whose principal function is to contribute to the formation of the voice, and prevent the entrance of aliments into the trachea, where they would necessarily occasion the most distressing symptoms. With these impressions it was natural to suppose that the want of it would induce a derangement of the functions of those parts, and that ultimately death would be the consequence; but I had an opportunity of making an observation which invalidates the truth of this opinion. I was preparing the parts concerned in the mechanism of the voice for the purpose of demonstration. The subject of my dissection was a man 58 years of age, who died in the hospital of an acute disease, without having experienced the smallest difficulty either in speaking or swallowing food. When I began to expose the parts to view, I was surprised to find the larynx *without an epiglottis.* The arytenoidei muscles were larger than common, and covered the glottis, as we see in many birds. On macerating the larynx, I distinctly perceived a cicatrix on the former seat of the epiglottis, from which it appeared evidently to have been removed by disease."

These observations are referred to merely as an historical fact, without attempting to depreciate the labours of Mr. Magendie, who has certainly the merit of having instituted a valuable series of experiments, by which the question has been brought brought to a satisfactory issue; and, admitting their faithfulness, I think the conclusions will never again be disputed.

It should also be remarked, that Tozzetti admits a fact mentioned by Severinus, which leads to an inference opposite to his own; and others of the same kind have been referred to by Haller, in his great work on Physiology. The patient whose case is related by Severinus lost his epiglottis in consequence of the venereal disease, and on attempting to swallow either solids or liquids, was almost suffocated. After his death the pericardium was discovered to be filled with water, and the lungs were of a livid colour.

